# Practical aspects of chimeric antigen receptor T-cell administration: From commercial to point-of-care manufacturing

**DOI:** 10.3389/fimmu.2022.1005457

**Published:** 2022-09-27

**Authors:** Nuria Martinez-Cibrian, Marta Español-Rego, Mariona Pascal, Julio Delgado, Valentín Ortiz-Maldonado

**Affiliations:** ^1^ Department of Haematology, Hospital Clínic de Barcelona, Barcelona, Spain; ^2^ Institut d’Investigacions Biomèdiques August Pi i Sunyer, Barcelona, Spain; ^3^ Department of Immunology, Hospital Clínic de Barcelona, Barcelona, Spain; ^4^ Department of Medicine, Universitat de Barcelona, Barcelona, Spain

**Keywords:** chimeric antigen receptor (CAR), cluster of differentiation 19 (CD19), point-of-care, B-cell, Non-Hodgkin's lymphoma (NHL), Acute lymphoblastic leukemia (ALL)

## Abstract

Chimeric antigen receptor T-cells targeting the CD19 antigen have achieved impressive results in patients with relapsed/refractory (R/R) B-cell malignancies, leading to their approval in the European Union and other jurisdictions. In Spain, the 100% academic anti-CD19 CART-cell product varnimcabtagene autoleucel (var-cel, ARI-0001 cells) has been extraordinarily approved under the Hospital Exemption clause for the treatment of patients older than 25 years of age with R/R acute lymphoblastic leukaemia. Var-cel has also been granted PRIority MEdicines designation by the European Medicines Agency for the same indication. In this review we reveal some practical aspects related to the preparation and administration of academic point-of-care CART-cell products, using var-cel as an example, and put them into the context of commercial products.

## Introduction

Chimeric antigen receptor (CAR) T cells targeting the CD19 antigen, such as tisagenlecleucel (tisa-cel) (1–3), axicabtagene ciloleucel (axi-cel) (4, 5), lisocabtagene maraleucel (liso-cel) (6) and brexucabtagene autoleucel (brexu-cel) (7) have shown impressive results in patients with relapsed/refractory (R/R) B-cell malignancies, leading to their approval in the European Union and other jurisdictions for the treatment of acute lymphoblastic leukaemia (ALL), diffuse large B-cell lymphoma (DLBCL) and other types of aggressive B-cell lymphoma, mantle-cell lymphoma (MCL) and follicular lymphoma (FL).

In Spain, the results from the CART19-BE-01 clinical trial evaluating the safety and efficacy of the 100% academic anti-CD19 CART-cell product varnimcabtagene autoleucel (var-cel, ARI-0001 cells) (8, 9), led to its extraordinary approval, as Hospital Exemption, by the Spanish Agency of Medicines (AEMPS) for patients older than 25 years of age with R/R ALL (10). This product has also been granted PRIority MEdicines (PRIME) designation by the European Medicines Agency for the same indication.

In this manuscript we review some practical aspects related to the preparation and administration of academic point-of-care (POC) CART-cell products and put them into the context of commercial products.

## Point-of-care vs. centralised manufacturing of CART-cells

Varnimcabtagene autoleucel (var-cel, ARI-0001 cells) was developed from scratch at our institution, starting from preclinical studies (11), scaling up of vector and cell production (12) and clinical trials (8, 9), all leading to the extraordinary approval for patients older than 25 years with R/R ALL (10) and a compassionate use programme. In our opinion, these milestones prove that it is feasible to manufacture advanced therapy medicinal products (ATMPs) in a median-size academic centre like Hospital Clínic of Barcelona. However, several issues, such as ensuring manufacturing quality and capacity, providing timely responses to referring clinicians, and ensuring representative (hopefully unbiased) patient inclusion, had to be tackled throughout this journey.

Entities manufacturing ATMPs must meet rigorous quality standards to achieve authorisation by regulatory entities, these requiring human, structural and financial resources. These standards have been traditionally considered impossible to attain by academic institutions. As a result, academic centres have focused on the development of new products, which were then sold to pharmaceutical companies with the purpose of bringing them to the patients. Indeed, many scholars wrongly assume that products coming from academic institutions are of less quality than the industrial ones and, accordingly, the burden of proof has always lied on academic CART-cell products, being forced to demonstrate that they are of equivalent quality as those produced at industrial scale. This not only involves the CART-cell product manufacturing, but also the speed of response from patient identification to product delivery.

To measure the manufacturing quality of any drug, it is important to estimate the manufacturing success (or failure) rate. Unfortunately, however, several CART-cell studies do not provide this information, and it is difficult to estimate what are “acceptable” rates of manufacturing success. This is of utmost importance for ATMPs manufactured in academic centres, particularly in those with POC manufacturing.

## Steps of the manufacturing process

**Figure 1:**
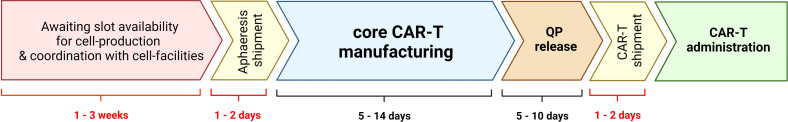
Steps required for autologous chimeric antigen receptor T-cell administration. Tentative timelines are added for reference. QP, qualified person.

Autologous CART-cell therapy, including all commercial products, follow a number of consecutive steps (**Figure 1**):

- Patient identification. This process may be fast if the patient is already linked with an accredited CART-cell centre, or lives in a region where accredited CART-cell centres exist, but may be significantly longer if the patient lives in a region without accredited centres. In this second scenario, appropriate authorisations from the patient’s own regional health care authorities must be sought, and payment secured (if needed), which could significantly delay the entire process. Finding affordable accommodation near the CART-cell centre is also becoming a very pressing matter for patients and their families.
- Signature of informed consent. Once the patient has arrived to the CART-cell centre, a number of medical tests (blood counts, chemistry, serologies, echocardiogram, pulmonary function tests, bone marrow aspirate, PET-CT scans, etc.) are needed to confirm that the patient remains a good candidate for therapy. Depending on the patient’s clinical situation, bridging therapy may be needed and planned, ideally after the cell collection.
- Cell collection by leukapheresis. Ideally, the cell collection should be linked to a production slot, but sometimes this is not possible and a cryopreservation step is needed before cell production. This situation typically occurs when the patient’s clinical condition requires urgent bridging therapy and this treatment may potentially compromise or delay T-cell collection.
- Cell production. This is the most “controlled” part of the process, lasting for 7-10 days for most CART-cell products. At the end of the production the cells are generally cryopreserved but, in some POC initiatives, they are administered fresh.
- Quality control (QC) tests. In the best case scenario, these tests may be completed in less than one day, including preliminary sterility tests (this would be the only way of administering a fresh CART-cell product to the patient). However, QC test results may take up to 5 weeks if some of these tests are outsourced (typically sterility tests) and, also, may be repeated if one or more results are not satisfactory or borderline satisfactory. Moreover, in case of manufacturing failure the entire procedure may be repeated, which would cause in a very significant delay for the patient.
- Cell infusion. This part of the procedure requires hospital admission and there may be delays caused by lack of hospital beds, concomitant infections in the patient or the community (e.g. COVID) or other medical problems. Moreover, preparative lymphodepleting chemotherapy, starting approximately one week before cell infusion, is normally planned once the product meets all the specification criteria. In some patients (e.g. those with severe neutropenia), hospital admission may be also required for lymphodepleting chemotherapy. Cells are normally infused all at once, but in some protocols (like ours), the cells are divided in three or four fractions. In our experience, this fractionated administration of CART-cells improves safety (8), but it also complicates the entire process, prolongs the hospital admission and does not allow for the administration of a fresh cell product (only the first fraction could potentially be administered fresh).

In summary, the manufacturing and infusion of CART-cells is a complex procedure that can be delayed for a number of reasons, like the ones mentioned above plus many others, such as medical complications affecting the patient (e.g. uncontrolled disease progression, unexpected infections or other medical complications, etc.) or logistical problems when the patients reside far from the accredited CART-cell centre.

In the last years, there has been an interest in the administration of fresh CART-cell products based on the assumption that cryopreservation (and thawing) can affect CART-cell functionality (13, 14). However, it must be stressed that fresh CART-cell products have a number of requirements:

- POC manufacturing or a manufacturing facility located in close proximity to the administration centre. Fresh CART-cell initiatives are not possible when the manufacturing unit is in a different city, country or continent.
- Single-dose administration of CART-cells. The fractionated administration of a fresh product is also impossible.
- Cell product release with preliminary sterility tests. Conventional microbiological cultures take too long for a fresh administration.
- Initiation of lymphodepleting chemotherapy before there is confirmation that there are enough CART-cells (or any CART-cells at all) in the final product. In case of a production failure, the patient may suffer from the complications of chemotherapy without any benefit from the cell infusion.
- Relatively stable baseline disease, since the procedure does not allow for unexpected medical complications (intervening infections, etc).

## Time to cell collection and the role of cryopreservation

As already stated, one important aspect of CART-cell manufacturing is the time required to prepare the patient for cell collection. As it may be obvious, considering the severity of the diseases at hand, the longer this process takes, the higher the probability of disease progression, medical complications and/or death.

Unfortunately, however, there is no consensus on how to define time to cell collection. For example, time to treatment can be defined as the time from informed consent signature (patient inclusion into the programme) to T-cell collection (leukapheresis). However, this may not be representative across the board since, for some products, informed consent signature only occurs when a leukapheresis/manufacturing slot is secured, whereas for other products informed consent signature is independent of manufacturing slot identification. In addition, T-cell collection is not necessarily related to the immediate initiation of cellular production since T-cells can be cryopreserved. Cryopreservation may be useful for patients with rapidly progressive disease in whom the administration of treatment is urgently needed, a treatment that could interfere with T cell collection. Thus, swift T-cell collection and cryopreservation before the initiation of salvage chemotherapy may ensure: i) the acquisition of sufficient and less heavily pretreated T-cells; and ii) the possibility to start CART-cell manufacture as soon as a free production slot is available, regardless of haematological recovery following treatment. Indeed, some commercial brands require cryopreserved T-cells and others cryopreserve the T-cells themselves at a later time point.

Our POC approach supports the use of either fresh or cryopreserved T-cells as starting material. Whenever possible, we favour the use of fresh T-cells because this allows for a prompt CART-cell manufacturing initiation within 24 hours of T-cell collection [73% of treated patients in the CART19-BE-01 trial (8)]. Moreover, the impact of cryopreservation on T-cell functionality is unclear (13, 14). It has been speculated that each cryopreservation process reduces T-cell functionality, but all commercial CART cell products are cryopreserved at least once or twice (15).

## Time of cell manufacturing

The time of cell manufacturing is an essential component of the time to treatment. This period can be divided in three parts: i) time required to transport the T-cell collection from the apheresis unit to the manufacturing facility; ii) time of cell culture and manipulation; iii) time of quality control tests before the product can be released; and (iv) time required to transport the manufactured product back to the patient.

The transportation time varies depending on the location of the manufacturing facility. In case of POC manufacturing, this time only represents a few minutes compared to days if the manufacturing facility is located in a different city, country or continent. Unfortunately, this time is not reported in pivotal trials evaluating commercial products, but can be estimated around 1-2 days for each trip.

Regarding the time of cell preparation (cell culture and gene manipulation), this is very difficult to modify once the manufacturing procedure is established and agreed with the regulatory agencies. The manufacture of var-cel lasts for a median of 8.5 days (range, 7-10 days), but this figure cannot be compared as this information is usually unavailable for other products. Some studies have reported a median cell production of 15 days for paediatric patients with ALL (16), whilst others have reported manufacturing and releasing times of 13 days for adult ALL (17), and 17 (18) and 24 (6) days for DLBCL.

## Vein-to-vein time

Although manufacturing/releasing times are usually less than 21 days, the time from cell collection (apheresis) to cell infusion [vein-to-vein (V2V) time] can be much longer. Moreover, the time from informed consent signature to infusion can be twice/thrice longer than the manufacturing/releasing time. For instance, in the CART19-BE-01 clinical trial the actual manufacturing time was 8.5 days, the V2V time was 42 days and time from informed consent signature to infusion was 54 days (8). These figures are comparable to the 45 days reported from study inclusion to cell infusion in paediatric patients with ALL treated with tisa-cel (1), or to the 53 days from informed consent signature to product arrival in paediatric patients with ALL treated with JCAR014 (16). Equivalent results have been published in adult patients with DLBCL, with a reported V2V time of 37 days (6), and 54 days from recruitment to treatment (2).

The reason why the V2V time is much longer than actual manufacturing time can be attributed to the compulsory quality control (QC) tests, which for var-cel include cell viability, purity, transduction efficiency, potency, and sterility (11). Cell viability, purity and transduction efficiency can be estimated within 24 hours by flow cytometry, but the quantification of CAR copies and the potency assay require a longer time (a minimum of 48 hours). In our experience, the major hurdle was related to sterility tests, which were initially outsourced, and for which definitive results were not available in less than 21 days. In a few occasions, these sterility tests took longer than 5 weeks. Currently all sterility checks are done at our own Microbiology Laboratory and, since this change was implemented, there has been a substantial reduction in the time required for all quality tests. Nowadays, the median time from the end of cell production to product release is 8 days. Put together, the median time for cell production initiation to product release is approximately 16 days.

On the other hand, the remaining QC tests (appearance, cell count, identity, purity, viability, presence of endotoxin/Mycoplasma/adventitious agents, vector copy number, potency assay and quantification of replication competent lentivirus) are easier to implement in a shorter time frame. Some of them are measured by flow cytometry, such as identity (percentage of CAR+ cells), purity (percentage of CD3+ cells) and the potency assay. The composition of the cell product is also routinely analysed by flow cytometry, including the percentages of T-cells (CD3/CD4/CD8), B-cells (CD19) and NK-cells (CD16/CD56).

Despite the median V2V time was 42 days for the CART19-BE-01 trial, which is in line with similar studies, we believe that it can be further reduced (besides improvements in QC tests). For instance, when we initiated our first clinical trial, we only had one bioreactor and therefore only two products could be elaborated per month. A second, third and fourth bioreactor became available in September 2017, May 2018, and February 2019, respectively, increasing our manufacturing capacity to approximately 8-12 products per month. Moreover, an increase in personnel at the Immunotherapy Unit also improved our V2V time. The availability of more production units and personnel has definitely allowed us to better adapt our cell productions to the patients’ needs, thus shortening waiting times and even reducing waiting lists.

Moreover, 83% of patients recruited into the CART19-BE-01 trial came from other Spanish regions, which added complexity in terms of logistics and administrative aspects. Although this is very difficult to calculate, this probably contributed to the 42 days V2V time, even though this is probably a common issue for other CART-cell programmes around the globe.

## Production failures

Among 54 patients recruited into the CART19-BE-01 trial, 47 patients eventually received var-cel therapy. The total number of manufacturing failures was 11, accounting for a 6% manufacturing failure rate (or 94% manufacturing success rate) (8). This is in line with other CART-cell products (**Table 1**) such as tisa-cel in paediatric and young adult R/R ALL (92.4%) (1) or in R/R DLBCL (93%) (2), and brexu-cel in R/R adult ALL (92%) (17).

**Table 1:** Manufacturing results and patients’ baseline characteristics for different CART-cell products targeting CD19 used for the treatment of relapsed/refractory B-cell malignancies.

	Manufacturing success rate	Screening failure rate	CART products not infused	Median lines of prior treatment	Prior exposure to		Prior alloHCT or autoHCT
					Blinatumomab	Inotuzumab	
Paediatric ALL (and young adults)							
Gardner et al. (16)	100%	NR	4%	3	15%	0	62%
Maude et al. (1)	92%	14%	18%	3	0	0	61%
Adult ALL							
Park et al. (19)	97%	6%	32%	3	25%	0	36%
Hay et al. (20)	NR	NR	10%	3			43%
Shah et al. (17)	91%	NR	23%	2	45%	22%	42%
Roddie et al. (21)	NR	3.8%	20%	3	25%	50%	65%
Adult DLBCL and other types of aggressive NHL							
Neelapu et al. (4) Locke et al. (18)	99%	NR	9%	3	–	–	21%
Schuster et al. (2)	93%	30%	33%	3	–	–	49%
Abramson et al. (6)	99%	NR	22%	3	–	–	35%
Paediatric and adult ALL and NHL							
Ortiz-Maldonado et al. (8)	94%	7%	13%	4	24%*	34%*	87%* (80% for both ALL/NHL)

*These percentages refer to ALL patients only.

alloHCT, allogeneic haematopoietic cell transplantation; autoHCT, autologous haematopoietic cell transplantation; ALL, acute lymphoblastic leukaemia; NHL, non-Hodgkin lymphoma; NR, not reported.

Among the 47 patients treated, seven (15%) required a repeated manufacturing procedure. In six patients, this was due to bacterial contamination of the product, and in one patient it was because the product did not meet all the specifications (the transduction efficiency was less than 20%). Among all 11 production failures, nine (82%) were caused by bacterial contamination. These contaminations happened in the first year of the trial. As more experienced was accumulated by our technical personnel, contaminations became rarer and have not happened anymore since 2019.

## How representative are patients treated with these novel agents?

Another important issue regarding CART-cells is how representative of daily practise are the patients enrolled in these programmes (**Table 1**). Restrictive inclusion and exclusion criteria can result in selecting patients with favourable conditions compared to most patients seen in clinics. The need to travel to a different region may also result in a bias towards patients with better socio-economical status or those fit enough to withstand travelling back and forth to the CART-cell institution.

Some variables can help us identify whether the patients included in a CART-cell programme are representative of patients seen routinely in clinic. These are: i) rate of screening failure; ii) proportion of patients who underwent apheresis but did not receive the product; iii) proportion of patients who signed the informed consent but did not received the product; and iv) prior treatment for the underlying disease.

Regarding screening failure rate, 93% (54/58) of patients who signed the consent form of CART19-BE-01 trial proceed to apheresis, and only 7% (4/58) were excluded at screening (8). This rate is similar to what has been reported in other studies. In AUTO1 trial 3.8% of adult patients with ALL were excluded (21), 6% of adult patients with ALL treated with brexu-cel (17) and 14% of paediatric B-ALL treated with tisa-cel (1) were also excluded at screening. Thirty percent of screening failures has been described in patients with DLBCL (2), whilst other studies only reported how many patients were recruited, not providing information on screening failures (6, 16–18). We consider that ≤ 10% of screening failure indicates that inclusion criteria were permissive and, therefore, patients treated were relatively representative of the true R/R ALL population. However, other factors may also play a role. In our CART19-BE-01 study, 2/4 patients who did not proceed to the apheresis withdrew consent because axi-cel had just been approved in the EU and these patients received this product in the context of an early access programme. Moreover, for many months our trial was the only CART-cell trial open in Spain for patients with R/R ALL, but this is no longer the case.

In the CART19-BE-01 trial, 47/54 (87%) patients who underwent leukapheresis eventually received var-cel. This 13% is in line with the 9.2% of products not infused in patients with DLBCL planned to receive axi-cel (4), 18% of children with ALL planned to receive tisa-cel (1), 20% of adults with ALL planned to receive AUTO1 (21), 21.8% of patients with DLBCL planned to receive liso-cel (6) and 22.5% of adults with ALL planned for brexu-cel therapy (17). Among the 7 patients from our study who did not receive the product, only one had an exclusion criterion (refractory and severe graft-versus-host disease). As for the other six patients, three did not receive the product due to manufacturing failure, one patient died before infusion, and two withdrew consent after the apheresis.

If we sum both, we can estimate the percentage of patients recruited but not treated in these studies, representing how strict patient selection was. In the CART19-BE-01 study, 58 patients signed consent form and 11 (19%) did not receive the product, which is similar to the 23% observed in AUTO1 trial (21), and probably compares favourably with the 30% reported in the ELIANA study (1), 36% in ZUMA-3 trial (17) and 46.6% in JULIET trial (2).

## The issue of prior therapy in CART-cell trials

The last crucial aspect in terms of patient representativity comes from the evaluation of prior therapy (**Table 1**). For patients with R/R ALL, in most cases prior therapy includes one (or more) allogeneic haematopoietic cell transplantation (alloHCT) (22). Moreover, new drugs such as blinatumomab (23) and inotuzumab (24) are now available in the EU for the treatment of R/R ALL, have improved the outcome of these patients per se, and have increased the percentage of patients referred for alloHCT. Indeed, current patients with R/R ALL treated in the EU and included in CART-cell studies should have been exposed to one or more of these agents/procedures, as is the case in clinical practise. In the CART19-BE-01 trial, patients with R/R ALL were exposed to a median of 4 previous lines of therapy at screening (8), in a similar way as in the ELIANA and AUTO1 trials (median of 3 prior lines) (1, 21). In contrast, patients recruited in the ZUMA-3 had received a median of 2 lines of therapy before inclusion in the trial (17), while other studies did not report this information. Since patients recruited in the CART19-BE-01 trial received more lines of prior treatment it could be argued that there is a potential higher risk of toxicity and/or disease progression, including clonal selection. Prior exposure to blinatumomab or inotuzumab may also raise some concerns regarding toxicity and efficacy. Lack of response to blinatumomab has been associated with reduced response rate and duration of response after CART-cells (25), whereas inotuzumab may be associated with an increased risk of sinusoidal obstruction syndrome (26).

Twenty-four percent of patients included in the CART19-BE-01 study received previous blinatumomab and 34% prior inotuzumab (8). Similarly, 25% and 50% of patients had been exposed to blinatumomab and inotuzumab, respectively, in the AUTO1 trial (21); whereas the equivalent figures were 45% and 22%, respectively, for the ZUMA-3 study (17). In children receiving treatment with JCAR014, 15.5% had been previously exposed to blinatumomab (16), and this percentage increased to 25% for adult patients receiving the same product (20). In the ELIANA study, there were no patients with prior treatment with blinatumomab or inotuzumab (1).

In addition, 87% of patients enrolled in CART19-BE-01 had already failed a prior alloHCT, this being among the highest percentage in CART-cell studies (8). Other CART-cell trials performed in adult ALL have reported that the percentage of patients with prior exposure to alloHCT was 65% (AUTO1) (21), 43% (JCAR014) (20), 42% (ZUMA-3) (17) and 36% (19). Regarding paediatric patients with R/R ALL, the use of alloHCT was reported in around 60% (JCAR014 and ELIANA) (1, 16).

## Immune monitoring of patients who received CART-cells

Another advantage of POC initiatives is the ease in which CAR+ and other immune cells can be identified and monitored in the laboratory. After the infusion of var-cel, patients at our institution are monitored twice weekly (first two weeks), then weekly until day +42, every two weeks until day +100 and monthly until day +365. The tests performed are: (i) T-B-NK flow cytometry panel to quantify the percentage of the different cell populations; (ii) CAR+-cell quantification by both flow cytometry (in percentage and absolute numbers) and quantitative PCR (copies of CAR construct per genome); (iii) human anti-murine antibodies, also by flow cytometry. The panel includes the following monoclonal antibodies: CD4-FITC, CD16/CD56-PE, CD8-PerCPCy5.5, CD19-PECy7, CD45-APC and CD3-BV421. The percentage of CAR+ cells is calculated using a specific monoclonal antibody against the A3B1-scFv conjugated with APC.

## Conclusions

The advent of CART-cells targeting CD19 has improved the outcome of patients with R/R B-cell malignancies. However, the high cost and logistic complications associated with centralised commercial CART-cell production have prompted the development of academic POC initiatives that, fulfilling the same strict quality criteria as commercial products, are able to achieve similar performance parameters such as manufacturing failure rate and vein-to-vein time. We hope that initiatives like ours can be replicated in other academic institutions around the globe, establishing themselves as valid alternatives to industrial CART-cell products.

## Author contributions

NM-C, VO-M, MP, and ME-R provided data. VO-M performed the comparison of var-cel results with published data. VO-M, NM-C, and JD wrote the first draft of the manuscript, that was approved by all authors. All authors contributed to the article and approved the submitted version.

## Acknowledgments

We are indebted to many colleagues who made this project possible. A non-exhaustive list includes Álvaro Urbano-Ispizua, Manel Juan, Maria Castellà, Josep Maria Canals, Jordi Esteve, Mercedes Montoro-Lorite, Gonzalo Calvo, Sara Varea, Toni Castells and Josep Maria Campistol. We are also very grateful to all the patients and their families who accepted to take part in our CART-cell programme.

## Conflict of interest

The authors declare that the research was conducted in the absence of any commercial or financial relationships that could be construed as a potential conflict of interest.

## Publisher’s note

All claims expressed in this article are solely those of the authors and do not necessarily represent those of their affiliated organizations, or those of the publisher, the editors and the reviewers. Any product that may be evaluated in this article, or claim that may be made by its manufacturer, is not guaranteed or endorsed by the publisher.
